# Alpine Treeline Dynamics and the Special Exposure Effect in the Hengduan Mountains

**DOI:** 10.3389/fpls.2022.861231

**Published:** 2022-04-08

**Authors:** Fuyan Zou, Chengyi Tu, Dongmei Liu, Chaoying Yang, Wenli Wang, Zhiming Zhang

**Affiliations:** ^1^Yunnan Key Laboratory of Plant Reproductive Adaptation and Evolutionary Ecology, School of Ecology and Environmental Sciences, Yunnan University, Kunming, China; ^2^Department of Environmental Science, Policy, and Management, University of California, Berkeley, Berkeley, CA, United States; ^3^Chinese Research Academy of Environmental Sciences, Beijing, China

**Keywords:** treeline dynamics, fraction vegetation cover, landscape scale, exposure effect, the Hengduan mountains

## Abstract

Alpine treeline is highly sensitive to climate change, but there remains a lack of research on the spatiotemporal heterogeneity of treeline and their relationships with climate change at the landscape scale. We extracted positions of alpine treeline from high-resolution Google Earth images from three periods (2000, 2010, and 2020) and analyzed the elevation patterns and dynamics of treeline positions in the Hengduan Mountains. Based on the treeline positions in 2020, a buffer zone of 300 m is established as the treeline transition zone, and the changing trend of the fraction vegetation cover (*FVC*) from 2000 to 2020 and its relationship with climate are also analyzed. Due to the special geographical and climatic environment, the treeline in the Hengduan Mountains area is high in the middle but lower in the surrounding areas. We found that over the past 20 years, the treeline position did not change significantly but that the *FVC* increased in 80.3% of the treeline areas. The increase in *FVC* was related to the decrease in precipitation in the growing season. The results also revealed a special exposure effect on the alpine treeline in the Hengduan Mountains. Because of the lower treeline, isotherm position caused by the monsoon climate, the treeline position on south-facing slopes is lower than that on slopes with other exposures. Our results confirmed that the pattern and dynamics of the alpine treeline are driven by the regional monsoon climate regime.

## Introduction

Alpine treeline is a sensitive indicator of the responses of terrestrial ecosystems to climate change (Lenoir et al., 2008; Hagedorn et al., 2019) and provides early warnings of global climate change (Alatalo and Ferrarini, 2017; Du et al., 2018). The treeline ecotone is expected to expand to higher latitudes and elevations because of rapid climate warming (Scuderi et al., 1993; Chen et al., 2011; Malanson, 2013). A global study showed that the treelines at only 52% of all 166 global treeline sites have advanced upslope over the past 100 years (Harsch et al., 2009). Recently, Lu et al. (2020) conducted a meta-analysis of the annual shift rate of treelines in the Northern Hemisphere and revealed that the treelines have shifted upward at 127 out of 143 sites (88.8%) since 1901. This shows that the trend of upward treeline movement is becoming increasingly obvious at most sites around the world. However, it has been suggested that the sensitivity of treeline ecotones depends more on changes in tree density than on the position of the treeline (Camarero and Gutierrez, 2004). Sigdel et al. (2020) quantified the changes in density and the spatial patterns of 17 treeline sites in the central Himalayas. The results suggested that younger trees showed clustering near the treeline. The method of historical reconstructions of tree positions has provided numerous examples of forest infilling in some alpine regions (Payette and Filion, 1985; Lyu et al., 2016; Wang et al., 2016). However, regardless of whether the treeline position shifts upward or the tree density increases (La Sorte and Jetz, 2010; Hagedorn et al., 2019; Rees et al., 2020), the area of tundra and meadow ecosystems at higher elevations will be reduced, resulting in the loss of biodiversity and ecosystem services (Thuiller et al., 2005; Feurdean et al., 2016; Pecl et al., 2017). Therefore, it is necessary to pay attention to these two aspects simultaneously while discussing the treeline pattern and its dynamic change.

Treeline exhibits a scale effect (Körner, 2012; Singh et al., 2015), which also determines the diversity of treeline research scales (Holtmeier and Broll, 2019). At the local scale, researchers usually focus on ecological processes, tree density changes, species distribution, and composition of the treeline transition zone (Liang et al., 2016; Sigdel et al., 2020). An increasing number of studies have analyzed position and spatial pattern changes in treeline on relatively large scales (global, regional, or landscape) (Guo et al., 2014; Mohapatra et al., 2019; Wei et al., 2020). However, there are differences in treeline patterns and their dynamics from local to global scales (Holtmeier and Broll, 2012; Marc and Johnson, 2013; Bader et al., 2020). For example, Gaire et al. (2014) found that the treeline in the central Nepal Himalaya is moving upward at a rate of 2.61 m/year at the sample plot scale. Mohapatra et al. (2019) found that the Himalayan treeline is moving upward at a rate of 11.3 m/year at the landscape scale. However, a meta-analysis of annual treeline shift rates in the Northern Hemisphere showed that the mean shift rate was only 0.354 m/year (Lu et al., 2020). This shows that many research results are controversial due to the influence of different scales.

How to choose an appropriate treeline scale to evaluate and predict the relationship between treeline change and climate change more comprehensively and accurately is a key problem. There are great differences among treelines in different climatic zones and biogeographic regions, but the influence of topographic factors on the spatial pattern of treeline is often ignored (Yirdaw et al., 2015; Bader et al., 2020). However, the treeline pattern on a fine scale is usually described as the relative position between trees (Sigdel et al., 2020), but this pattern cannot be applied to other areas. In addition, different scales will also affect the response of treeline to climate. On a fine scale, physical and biological factors may interfere with the response of tree growth to climate (Hellmann et al., 2016), and the position of treeline might be inconsistent with the climate conditions (Marc and Johnson, 2013). On the global scale, local differences will be ignored, and it may be easy to overemphasize coarse drivers such as temperature (Harsch et al., 2009; Moyes et al., 2015). The landscape scale may better reflect the relationship between treeline dynamics and climate (Batllori and Gutiérrez, 2008; Case and Duncan, 2014; Singh et al., 2015). However, how to obtain large-scale and high-precision treeline data is also a problem to be solved in the current research.

The Hengduan Mountains provide an extensive longitudinal, latitudinal, and topographical framework for studying the landscape pattern and dynamics of treeline. The objective of this study was to characterize the responses of treeline dynamics (treeline position and vegetation cover) to changes in climate at the landscape scale. Specifically, high-resolution Google Earth images were used to extract the alpine treeline in the Hengduan Mountains area in different periods, and then the changes in treeline position and vegetation coverage, and also their relationships with climate, were assessed. The results of this study provide new insights into the factors driving treeline dynamics at the landscape scale, and these insights could aid in the evaluation of the risks faced by alpine ecosystems and species in the alpine regions under future climate change.

## Materials and Methods

### Study Area

The Hengduan Mountains (24°39′N–33^°^34′N, 96°58′E–104°27′E) are composed of a series of north–south-trending parallel mountains and rivers (Li, 1987). The terrain of this region fluctuates greatly, with high terrain in the north and low terrain in the south; there are many high mountains and deep valleys (Wen, 1989). The climate conditions are complex, and the temperature and precipitation in the region vary greatly (Bai et al., 2019). The mean annual temperature ranges from 5 to 13°C, and the total annual precipitation ranges from 500 to 1,000 mm (Dai et al., 2020). Since the 1960s, the mean annual temperature of the Hengduan Mountains area has increased, and the rate of increase has increased significantly since 2000 (Li et al., 2010). This region is also one of the most biodiverse regions worldwide, with high species diversity and endemism (Myers et al., 2000; Sun et al., 2017). Natural treelines are abundant and are ideal for studying the dynamic responses of treelines to climate (Miehe et al., 2007; Kramer et al., 2010).

**FIGURE 1:**
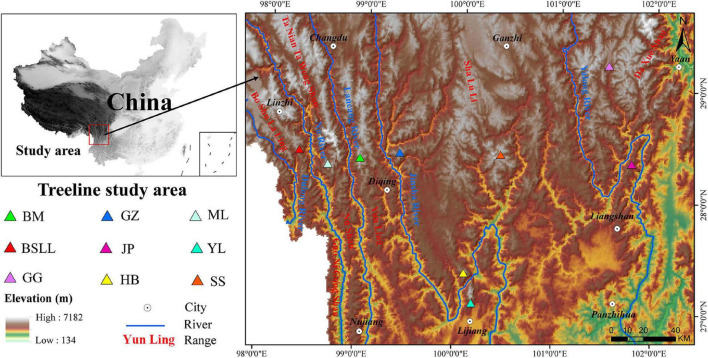
Location of the studied treeline sites in Hengduan Mountains area. BSLL, Boshulaling Mountain; ML, Meili Snow Mountain; BM, Baima Snow Mountain; GZ, Gezong Snow Mountain; HB, Haba Snow Mountain; GG, Gongga Mountain; SS, Sanshen Mountain; YL, Yulong Snow Mountain; JP, Jinping Mountain.

The forest ecosystem in the Hengduan Mountains is mainly distributed in the south. We choose 9 mountains at different longitudes and latitudes in this region (Figure 1), each of which features a large number of undisturbed treelines. The nine main mountains are the Boshulaling Mountain (BSLL), Meili Snow Mountain (ML), Baima Snow Mountain (BM), Gezong Snow Mountain (GZ), Haba Snow Mountain (HB), Gongga Mountain (GG), Sanshen Mountain (SS), Yulong Snow Mountain (YL), and Jinping Mountain (JP). These mountains are national nature reserves, provincial nature reserves, or major mountains in the region.

### Extraction of the Treeline

**FIGURE 2:**
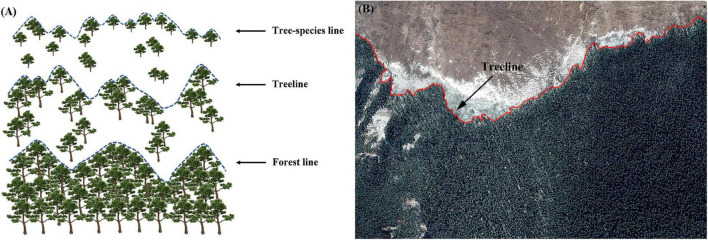
**(A)** The position of treeline. **(B)** Extraction of treeline from Google Earth images.

Generally, the alpine treeline is located between the tree species line (upper limit of seedlings or young trees) and the forest line (upper limit of closed forest) and roughly corresponds to the line linking the densest patches of trees >3 m in height (Körner, 2012; Singh et al., 2015), as shown in Figure 2A.

In this study, the alpine treeline is defined as the line representing the physical boundary between forest and alpine vegetation. Treeline data were extracted using remote-sensing images in Google Earth software (Figure 2B). Elevation is an important variable for distinguishing the climatic treeline from a disturbance treeline (Holtmeier and Broll, 2005; Leonelli et al., 2013). According to the previous research, the climatic treeline in the study area is greater than 3,800 m (Wang et al., 2013). However, the elevation of disturbed treelines is often lower than that of climatic treelines (Holtmeier and Broll, 2005; Leonelli et al., 2013). To account for the effect of interference factors on the obtained treeline data, the canopy boundary formed by natural interference factors, such as topography, snow cover, landslides, and gullies and human interference factors, including road construction, mining, and grazing, was removed. Google Earth images from 2000, 2010, and 2020 were used to extract treeline data, and the treeline data obtained from Google Earth were imported into ArcGIS software and converted into grid data.

### Extraction of the Fraction Vegetation Cover

#### Extraction of Normalized Difference Vegetation Index

Landsat images with 30-m resolution in the study area were used to extract the normalized difference vegetation index (NDVI), and then the vegetation coverage was extracted (Wilson and Norman, 2018). The formula is as follows:

$$\mathrm{NDVI}=\frac{\rho_{\mathrm{NIR}}-\rho_{\mathrm{RED}}}{\rho_{\mathrm{NIR}}+\rho_{\mathrm{RED}}}$$

where *ρ_*NIR*_* is the reflectivity of the near infrared band and *ρ_*RED*_* is the reflectivity of visible light in the red band.

#### Calculation of the Fraction Vegetation Cover

The fraction vegetation cover (FVC) was calculated based on the NDVI (Gu et al., 2009), and the formula is as follows:

$$\mathrm{FVC}=\frac{\mathrm{NDVI}-{\mathrm{NDVI}}_{\min}}{{\mathrm{NDVI}}_{\max}-{\mathrm{NDVI}}_{\min}}$$

where NDVI_*min*_ is the minimum value of the vegetation index, which is the NDVI value of bare or non-vegetated areas on the remote sensing images, and NDVI_*max*_ is the maximum value of the vegetation index, which is the NDVI value of an area completely covered by vegetation, that is, the NDVI value of pure vegetation pixels.

### Extraction of Climatic and Topographic Factors

#### Climate Data

Climate data with a 1-km resolution were downloaded from the monthly precipitation and temperature data of China from 1961 to 2020 from the National System Earth Science Data Center, National Science and Technology Infrastructure of China.^1^ Then, the monthly climate grid data were clipped, projected, and calculated by ArcGIS software to obtain gridded data of spring mean temperature, summer mean temperature, autumn mean temperature, winter mean temperature, spring precipitation, summer precipitation, autumn precipitation, and winter precipitation in the study area.

The precipitation and mean temperature of the growing season (PGS and MTGS) were extracted from the monthly precipitation and mean temperature layer data following the method of Wang et al. (2013). The mean temperature of the growing season was defined as the mean value of the monthly mean temperature ≥5°C (Walther and Linderholm, 2006). The raster calculator tool in ArcGIS was used for processing; the formula is as follows:

$$\mathrm{MTGS}=\frac{\sum_{1}^{12}T_{i}\geq 5\times F\,l\,o\,a\,t\,\left({\text{T}}_{i}\right)}{\sum_{1}^{12}{\text{T}}_{i}\geq 5}$$

$$\mathrm{PGS}=\frac{\sum_{1}^{12}P_{i}\times \sum_{1}^{12}T_{i}\geq 5}{\sum_{1}^{12}{\text{T}}_{i}\geq 5}$$

where *Ti* and *Pi* refer to the mean monthly temperatures, and the float (*Ti*) function represents the conversion factor for converting integer data to floating-point data.

#### Geographic Data

The Hengduan Mountains is located in the southeastern part of the Qinghai-Tibet Plateau, and its topography is extremely complex. Terrain factors were considered in the analysis of the treeline formation mechanism, and digital elevation data with 12.5-m resolution was used.^2^ The surface tools in ArcGIS were used to extract aspect and slope data.

### Data Analysis

The Extract Multi Values to Points module in the ArcGIS Spatial Analyst toolbox was then used to associate the treeline data with temperature, precipitation, aspect, and other information. A total of 777,649 treeline grids were obtained (Table 1), and then Excel was used to export the data for data analysis. The average alpine treeline distribution at different latitudes, longitudes, slope angles and aspects, and the proportions of the treeline on different slopes in the Hengduan Mountains area were analyzed.

**TABLE 1:** Numerical statistics of treeline grids in each slope direction.

Aspect	Count	2000		2010		2020	
		Elevation ± SD (m)	MTGS ± SD (°C)	Elevation ± SD (m)	MTGS ± SD (°C)	Elevation ± SD (m)	MTGS ± SD (°C)
N	81416	4205 ± 140	7.49 ± 0.93	4208 ± 144	7.45 ± 0.93	4212 ± 144	7.44 ± 0.94
NE	100548	4194 ± 148	7.47 ± 0.97	4199 ± 152	7.47 ± 0.97	4203 ± 151	7.45 ± 0.97
E	107981	4153 ± 137	7.57 ± 0.98	4165 ± 139	7.54 ± 0.98	4168 ± 139	7.52 ± 0.99
SE	100713	4127 ± 130	7.61 ± 0.97	4132 ± 135	7.58 ± 0.97	4137 ± 135	7.58 ± 0.97
S	94092	4132 ± 138	7.56 ± 0.95	4136 ± 140	7.57 ± 0.95	4143 ± 141	7.54 ± 0.96
SW	109680	4128 ± 139	7.69 ± 0.89	4130 ± 139	7.69 ± 0.89	4138 ± 141	7.67 ± 0.9
W	102017	4152 ± 138	7.75 ± 0.85	4155 ± 136	7.73 ± 0.85	4164 ± 137	7.72 ± 0.85
NW	81202	4185 ± 141	7.68 ± 0.88	4188 ± 143	7.66 ± 0.88	4198 ± 142	7.62 ± 0.88

The factors potentially affecting the elevation of the treeline were analyzed using convergent cross mapping (CCM). The basic idea of CCM is to use prediction between variables as a test for causality. If variable X has a causal effect on variable Y, then causal information of variable X should be present in Y; thus, the attractor recovered for variable Y should be able to predict the states of variable X. In practice, the Pearson correlation coefficient between the original time series Y and its estimate from the CCM by another time series X is used as a criterion for establishing causality (ρ_*ccm*_). ρ_*ccm*_ is positively related to the degree to which X can reconstruct Y; thus, the causal effect is stronger as ρ_*ccm*_ increases (Sugihara et al., 2012; Lin et al., 2020). The focus of our study was on a more specific problem: distinguishing the effects (causes) of variation in the elevation of treeline from different types of climatic factors. We used a spatial version of CCM to detect the causality between the response variable (result) and putative drivers (Table 2). For each possible pair (one putative variable and one response variable), we randomly permuted the indices of the paired variables and then calculated their correlations with ρ_*ccm*_. If the correlation withρ_*ccm*_→1, then the climatic variable is a driver of treeline elevation; if ρ_*ccm*_→0, then the climatic variable is not causally related to treeline elevation. The shuffling procedure was repeated 100 times. A Wilcoxon signed-rank test was used to determine whether the median was significantly greater than zero. A small *p*-value suggests that it is unlikely that the environmental variable affects treeline elevation. The analysis was carried out in R 3.6.1.

**TABLE 2:** Variables used for the impact factors analysis.

Variable types	Abbreviation	Full name
Climatic variables	MGTS	Mean temperature of growing season
	WinterMT	Winter mean temperature
	AutumnMT	Autumn mean temperature
	SummerMT	Summer mean temperature
	SpringMT	Spring mean temperature
	PGS	Precipitation of growing season
	WinterP	Winter precipitation
	AutumnP	Autumn precipitation
	SummerP	Summer precipitation
	SpringP	Spring precipitation
Terrain variables	—	Slope
	—	Elevation
	—	Longitude
	—	Latitude

The Theil-Sen median trend analysis method is a robust non-parametric statistical trend calculation method (Sen and Kumar, 1968; Theil, 1992), and its formula can be written as follows:

$$S_{\mathrm{FVC}}=\mathrm{median}\,\left(\frac{{\mathrm{FVC}}_{j}-{\mathrm{FVC}}_{i}}{j-i}\right),2000\leq i<j\leq 2020;$$

*S*_*FVC*_, which is used to quantify a monotonic trend, is the median of the slope of the *n*(*n*-1)/2 data combinations, and FVC*_*j*_* and FVC*_*i*_* represent the FVC values in years *i* and *j*. When *S*FVC >0, the *FVC* of the time series shows an increasing trend; when *S*_*FVC*_ <0, the FVC of the time series shows a decreasing trend.

The Mann–Kendall test (Kendall, 1938; Mann, 1945) is a non-parametric statistical test that measures the significance of a trend. When the Mann-Kendall method is applied to the *FVC* trend, the value of a certain time series is regarded as a set of independently distributed sample data, and the parameter *Xc* is used as the pixel *FVC* attenuation index. The calculation formula is as follows:

$$X_{c}=\left\{ \begin{array}{cc} \frac{M-1}{\sqrt{\mathrm{var}\,\left(M\right)}}\; & M>0\\ 0\; & M=0\\ \frac{M+1}{\sqrt{\mathrm{var}\,\left(M\right)}}\; & M<0 \end{array}\right.$$

Where

$$M=\sum_{i=1}^{n-1}\sum_{k=i+1}^{n}\mathrm{sign}\,\left({\mathrm{FVC}}_{k}-{\mathrm{FVC}}_{i}\right)$$

$$v\,a\,r\,\left(M\right)=\frac{n\,\left(n-1\right)\,\left(2\,n+5\right)}{18}$$

$$\mathrm{sign}\,\left({\mathrm{FVC}}_{k}-{\mathrm{FVC}}_{i}\right)=\left\{ \begin{array}{cc} 1\; & {\mathrm{FVC}}_{k}-{\mathrm{FVC}}_{i}>0\\ 0\; & {\mathrm{FVC}}_{k}-{\mathrm{FVC}}_{i}=0\\ -1\; & {\mathrm{FVC}}_{k}-{\mathrm{FVC}}_{i}<0 \end{array}\right.$$

Where FVC*_*k*_* and FVC*_*i*_* are sample time series datasets, *n* is the dataset length, and sign is a symbol function. At a given significance level *a*, when *| Xc |* > μ_1_-a/2, the time series data of the study represents a significant change in the α level, where ± X_1_-a/2 is the standard normal deviation. In this study, we take *a* = 0.05, and when *| Xc* | ≥ 1.96, the time series confidence level is *a* < 0.05 and *vice versa*. We not only use Theil Sen median trend analysis to test the change of fraction vegetation cover, but also make corresponding analysis on temperature and precipitation, and obtain *S*_*FVC*_, *S*_*tem*_, and *S*_*pre*_ values, respectively.

We calculated the Pearson correlation coefficient among temperature change, precipitation change, and FVC change. The Pearson partial correlation coefficient reflects the relationship between one climate factor and vegetation coverage after controlling for the influence of another climate factor (after eliminating the influence of this factor). According to the difference between the correlation coefficient and partial correlation coefficient, the correlation degree among temperature change, precipitation change, and vegetation coverage change is described.

## Results

### Treeline Elevation

**FIGURE 3:**
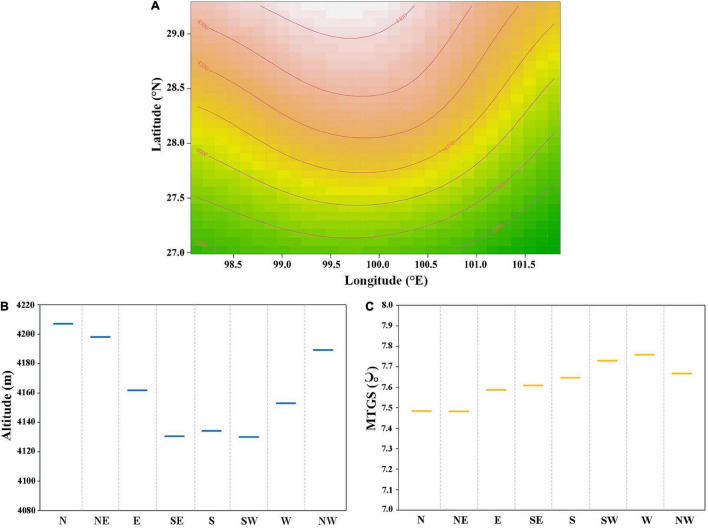
**(A)** Variation of treeline elevation with longitude and latitude, orange lines represent treeline elevation contours; **(B)** average elevation of alpine treeline with different slope directions; **(C)** mean temperature of growing season of alpine treeline with different slope directions.

The distribution of alpine treeline in the region spans approximately 4° of longitude (98.1–101.7°E) and approximately 3° of latitude (27–29.2°N) (Figure 3A). The treeline elevation in high-latitude areas is higher than that in low-latitude areas, and the treeline elevation in the middle is higher than that in the east and west. The treeline elevation difference reaches 600 m. Overall, the elevation of alpine treeline in the region is higher in the middle and lower in the surrounding areas.

The distribution of the treeline is also affected by the aspect. The treeline elevation is significantly lower on southern, southeastern, and southwestern slopes than on northern, northeastern, and northwestern slopes, and the average aspect-dependent elevation difference reaches 84 m (Figure 3B). However, there is little difference in temperature in the growing season among the different aspects (Figure 3C), which indicates that the average temperature isotherm in the growing season of the treeline on south-facing slopes is lower than that on slopes with other aspects.

### Impact Factors for Treeline Elevation and the Fraction Vegetation Cover

**FIGURE 4:**
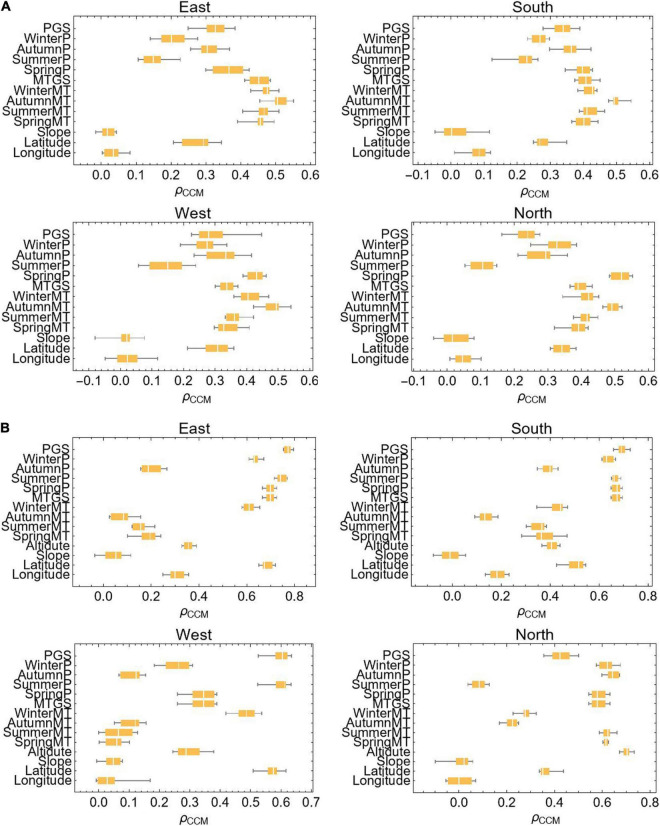
Causality test of factors affecting treeline elevation. **(A)** Treeline elevation; **(B)** the fraction vegetation cover; ρ_*CCM*_ represents the strength of causality.

To facilitate the analysis, we combined the eight slope directions with few differences in treeline elevation, and divided them into four slope directions (east, south, west, and north). Because climatic factors such as temperature and precipitation vary in their effects on the treeline elevation, the relative importance of these factors needs to be parsed carefully. We thus subdivided climatic and terrain factors into several categories to explore their relative importance in determining treeline elevation (Table 2). Generally, climatic factors were more important than terrain factors in predicting treeline elevation (Figure 4A). Among the various climatic variables, temperature was the most important (*ρ_*ccm*_* > 0.5). However, there was a weak linear relationship or no relationship between treeline elevation and various terrain factors.

Similarly, to understand the potential main driving factors of vegetation coverage in the treeline transition zone, we also use the corresponding methods for analysis, where the terrain factor is added to the elevation. When predicting vegetation coverage, the influencing factors of different slope directions vary (Figure 4B). On the east slope, south slope, and west slope, the key factor affecting vegetation cover is precipitation (*ρ_*ccm*_* > 0.7, *ρ_*ccm*_* > 0.7, and *ρ_*ccm*_* > 0.6), and on the north slope, in addition to precipitation, elevation is also an important factor (*ρ_*ccm*_* > 0.7).

### Treeline Dynamics and Climate Change

#### Treeline Elevation Change and Climate Change

**FIGURE 5:**
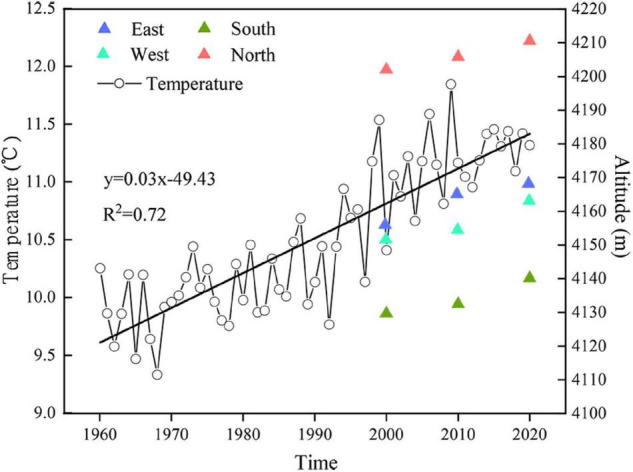
Relationship between temperature change and treeline shift.

The relationships between different temperature variables and treeline elevation in 2000, 2010, and 2020 are shown in Figure 5. From 2000 to 2020, the temperature increased, and the treeline shifted. The elevations of treelines on the east, south, west, and north slopes shifted by 12, 10, 1, and 8 m, respectively. Since the resolution of the digital elevation model (DEM) data used in the study area is 12.5 m and the change in treeline elevation is within this resolution range, we believe that there has been no significant change in the treeline elevation over the last 20 years.

#### The Fraction Vegetation Cover Change and Climate Change

**FIGURE 6:**
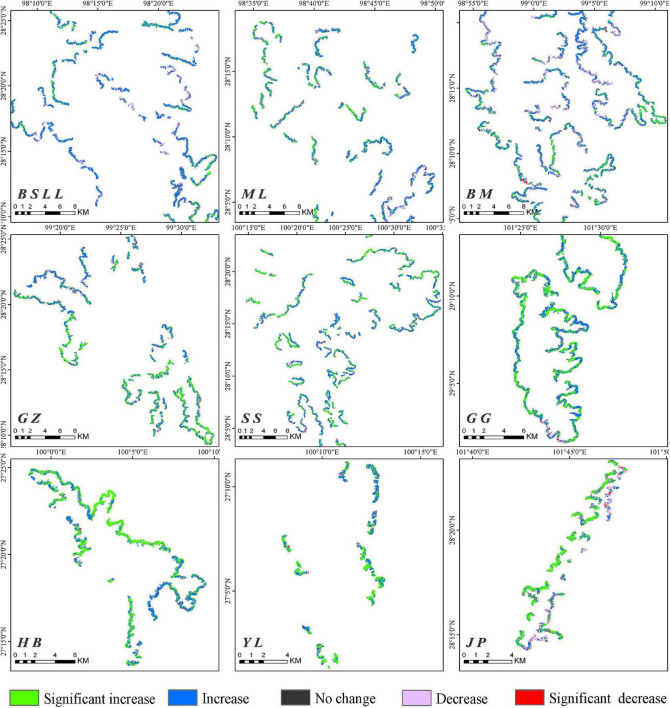
Spatial variation trend analysis of fraction vegetation cover on the main mountains in the Hengduan Mountains from 2000 to 2020.

Based on the FVC raster data from 2000 to 2020, the 21-year FVC of each pixel and the slope (S_*FVC*_) with significance were calculated. The purpose was to illustrate the spatial distribution of the FVC in the treeline transition zone and the characteristics of the spatial changes over time. In ArcGIS software, we used the buffer zone established by the treeline, and then the change trend of the FVC in the treeline transition zone was obtained (Figure 6).

**FIGURE 7:**
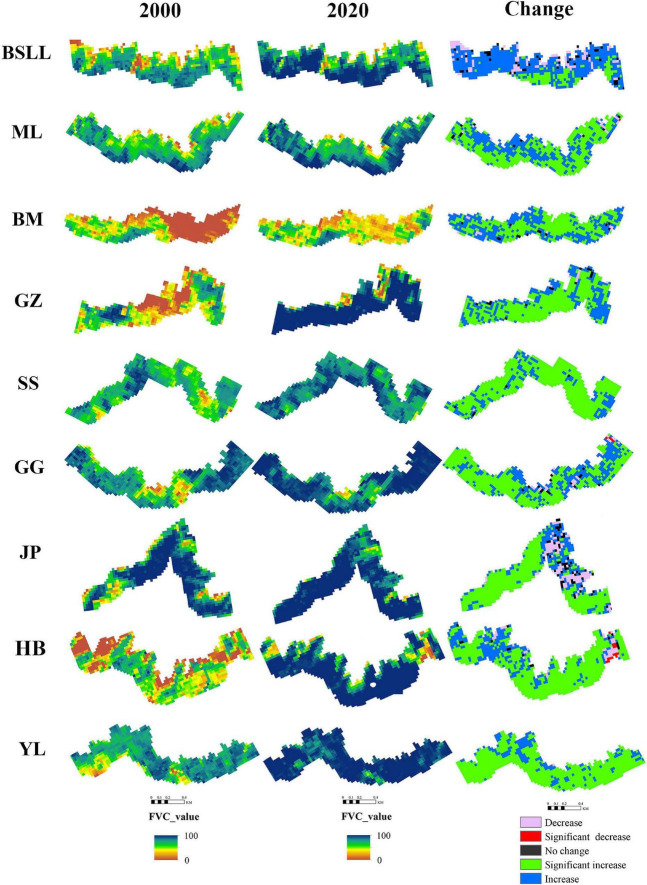
Changes of the fraction vegetation cover in parts of each mountain range.

A comparison of 2000 and 2020 shows that the FVC in the treeline transition zone of each mountain range increased significantly (Figure 7). Overall, during 2000–2020, the *increase in FVC* in the treeline transition zone (80.30%) was much larger than the decrease (13.37%), with areas of significant increases, slight increases, significant decreases, slight decreases and no changes accounting for 30.17, 50.13, 0.69, 12.68, and 6.33%, respectively (Table 3). These results indicate that the tree density in the *treeline transition zone* has significantly increased in the last 20 years.

**TABLE 3:** Statistical analysis results of *FVC* trends in treeline transition zone.

Slope of *FVC*	Significance	*FVC* trend	Area percentage (/%)
*S*_*FVC*_ > 0	*P* < 0.05	Significant increase	30.17
*S*_*FVC*_ > 0	*P* > 0.05	Increase	50.13
*S*_*FVC*_ = 0	—	No change	6.33
*S*_*FVC*_ < 0	*P* < 0.05	Significant decrease	0.69
*S*_*FVC*_ < 0	*P* > 0.05	Decrease	12.68

**FIGURE 8:**
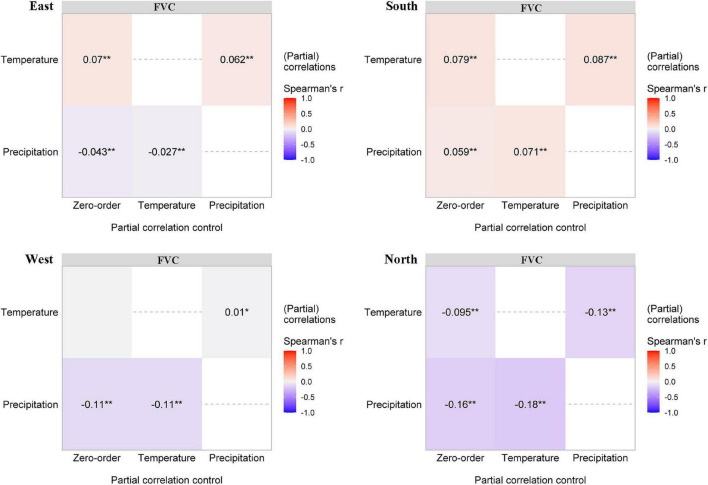
Partial correlations (Pearson’s *r*) between fraction vegetation cover and the two climatic variables. The intensity of colors and numbers indicate the strength of the correlation. Significant levels are: *P < 0.05; **P < 0.01.

The difference between zero-order and partial correlations indicated the degree of dependence of the correlation between the climatic variables and the FVC (Figure 8). The dependence between one climatic factor and vegetation coverage changed after removing the effect of another climatic factor; for example, temperature affects the relationship between precipitation and FVC change. The FVC change associated with different slope directions is significantly affected by temperature and precipitation. The dependence degree between FVC change and temperature change on the east and south slopes (Pearson’s *r* = 0.062 and 0.087, *P* < 0.01) is higher than that between FVC change and precipitation change (Pearson’s *r* = −0.027 and 0.071, *P* < 0.01). However, the dependence degree between FVC change and temperature change and precipitation change on the west and north slopes (Pearson’s *r* = 0.062 and 0.087, *P* < 0.01) is significantly higher than that between FVC change and temperature change alone (Pearson’s *r* = −0.027 and 0.071, *P* < 0.01).

## Discussion

The treeline elevation in the Hengduan Mountains area is high in the middle but lower in the surrounding areas. This may be caused by the mass elevation effect and the unique monsoon climate. Compared with the edges of the mountain at similar elevations, the interior part of the mountain range experiences higher solar radiation input and higher temperatures (Holtmeier, 2009; Han et al., 2020). This temperature difference is consistent with the difference in the elevation of the treeline between the inner and outer parts of the mountain range (Wang et al., 2017). The study area is also affected by the southwestern monsoon and the southeastern monsoon; precipitation decreases from southwest to northeast and from southeast to northwest (Li et al., 2010; Yao et al., 2012). The elevation of the treeline also increases along the direction of seasonal winds (Wang et al., 2013; Yao and Zhang, 2015).

**FIGURE 9:**
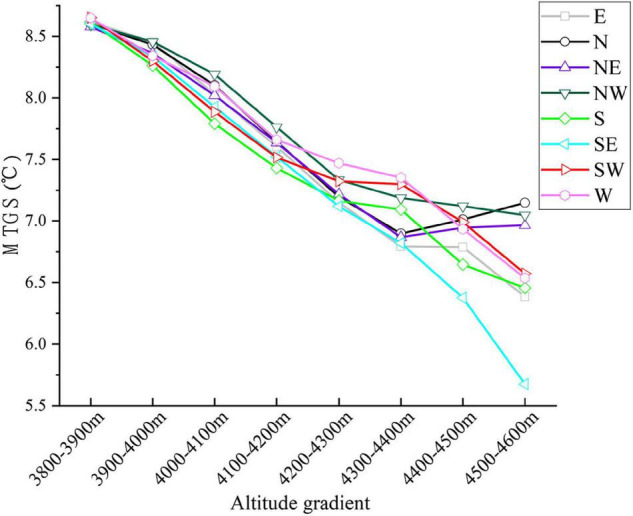
The variation of mean temperature in growing season along the elevation gradient of treeline in different slope directions.

In general, the treeline can reach greater heights on warm southern slopes compared with cold northern slopes (Malyshev and Nimis, 1997). Our study found that the elevation of the treeline is generally lower on the southern slope of the Hengduan Mountains than on the northern slope, but there is no difference in the mean temperature during the growing season. This shows that the growing season temperature at the treeline is consistent, while the isotherm distribution of the treeline on the southern slope is lower than that on the slopes with other aspects. To further support our conclusion, we calculated statistics for the mean growing season temperature of different aspects at different elevations and found that the mean growing season temperature of the southern slope at a given elevation is lower than that of the northern slope (Figure 9). These findings are consistent with a study in the Mediterranean region showing that treeline elevation on southern slopes may be limited by summer drought, which reduces the elevation of the treeline (Piper et al., 2016; Bonanomi et al., 2018). Different from the previous conclusions, the low elevation of the treeline on the southern slope of the Hengduan Mountains is not caused by drought but by the low temperatures induced by the special regional monsoon climate. This stems from the formation of a large number of clouds on the southern and western slopes of the Hengduan Mountains after noon, and the blocking of solar radiation and the low temperatures caused by rainfall decrease the elevation of the treeline (Poveda et al., 2005; Körner, 2012; Wang et al., 2013). In addition, the south-facing slopes are steeper and barren, making it very difficult for seedlings to colonize and grow.

Treeline location has a time lag in terms of reflecting climate (Holtmeier and Broll, 2019). In this study, we calculated the multiyear mean seasonal temperature to analyze the relationships between treeline elevation and various climate factors. Since there are obvious differences in the treeline elevation among each aspect, we use CCM to test the causal relationships between the treeline elevation of each aspect and climate variables and topographic variables. Compared with topography and precipitation, temperature has a stronger driving effect on treeline elevation. Although the range of the temperature threshold for tree growth is not clearly defined, it is widely accepted that the treeline position is closely related to temperature (Case and Duncan, 2014). The upper limit of the treeline is thought to be determined by the effect of temperature on the ability of trees to form new tissue rather than by a lack of photosynthesis (Körner, 1998). Both biogeographic and physiological evidence strongly suggests that low temperatures are the most important factor limiting the upslope advance of the treeline (Cieraad, 2011; Case and Duncan, 2014). Temperature is a critical factor limiting the production and differentiation of xylem cells in cold climates, and thermal conditions below a certain temperature can inhibit xylem growth (Rossi et al., 2008). Immature trees are unable to survive in alpine areas with low air temperatures (Li and Yang, 2004). Thus, under undisturbed natural conditions, as long as there is soil and sufficient moisture, trees can grow until a low temperature limit is reached, forming a natural climatic treeline (Körner, 2020).

Due to the impact of global warming, the growth of trees in most treeline sites has responded positively to the rise in temperature (Harsch et al., 2009; Camarero et al., 2021). A temperature increase gradually alleviates physiological limitations associated with low temperatures in alpine trees (Körner and Paulsen, 2004; Pandey et al., 2020). Most global treeline advances correspond to strong winter warming (Harsch et al., 2009). Due to the obvious differences in the degree of seasonal warming experienced by different regions, the treeline position is greatly affected by the local climatic and topographic conditions (Armbruster et al., 2007; Bonanomi et al., 2020). Since 1960, the temperature in the Hengduan Mountains has increased at a rate of 0.03°C/year, and in recent decades, the temperature has increased by approximately 1.8°C. According to the IPCC (2018) assessment report, the global temperature has increased by nearly 1°C during the past century (1906–2017). Although the speed of the temperature increase in the Hengduan Mountains area is much higher than the global average, the position of the treeline has not obviously moved upward. Based on a previous treeline study in the central Hengduan Mountains, the larch and fir trees required averages of 18 ± 2 (*n* = 150) and 33 ± 5 (*n* = 30) years to reach 2 m in height, respectively (Wang et al., 2019). This means that a seedling may require 27–50 years to reach 3 m in height (treeline trees), so 20 years is still too short to recognize a treeline advance.

Although there was no obvious upward trend in treeline elevation in the Hengduan Mountains over the past 20 years, the trend of increasing vegetation coverage was significant. In this study, 80.3% of the regional FVC showed an upward trend, of which 30.17% showed a significant upward trend. The FVC was strongly affected by precipitation in the growing season. A warm-growing season and adequate moisture are favorable for seedling emergence and growth (Körner, 2012; Han et al., 2018). In some drought-prone areas, tree recruitment also showed a gradual decrease in the treeline transition zone (Camarero and Gutierrez, 2004; Liang et al., 2014). However, our study shows that the increase in the FVC is related to a decrease in precipitation. Because precipitation decreases with increasing elevation, considering the impact of water availability (Liang et al., 2014), trees may not become established at higher elevations that are seasonally dry. In this context, trees tend to gather (Moyes et al., 2015; Treml and Chuman, 2018). Sigdel et al. (2020) showed a higher clustering tendency in dry treelines than in wet treelines. In high-elevation areas with high abiotic stress, plants can have a positive impact on each other, reduce the negative impact of adverse environmental conditions and improve the species richness of adjacent areas (Callaway et al., 2002; Smith et al., 2003; Chen et al., 2020). The FVC in 13.37% of the treeline areas showed a decreasing trend, and only 0.69% showed a significant decrease. Although this study carefully removed disturbed treeline areas in the process of treeline extraction in the early stage, the damage to vegetation in the alpine areas caused by natural interference factors (avalanches, landslides, gullies, etc.) and human interference factors (road construction, mining, grazing, fire, etc.) cannot be ignored (Ameztegui et al., 2016; Holtmeier and Broll, 2019; Wang et al., 2019).

## Conclusion

The rate of the temperature increase in the Hengduan Mountains is much higher than the global average. Under this background, the treeline position has not changed significantly, but the FVC in the treeline area has markedly increased. The results also revealed a special exposure effect of alpine treeline in the Hengduan Mountains. Because of the lower treeline isotherm position caused by the monsoon climate, the treeline position on south-facing slopes is lower than that on the slopes with other exposures. The aforementioned results are helpful for understanding the dynamic changes in treeline under the background of climate warming at the landscape scale and provide a reference for predicting and evaluating the threats faced by alpine ecosystems in the future.

## Data Availability Statement

The original contributions presented in the study are included in the article/supplementary material, further inquiries can be directed to the corresponding author.

## Author Contributions

WW designed the study. FZ and CY collected and processed data. CT and FZ conducted the statistical analyses. FZ wrote the manuscript. ZZ and DL revised the manuscript. All authors commented upon and contributed to the final version of the manuscript.

## Conflict of Interest

The authors declare that the research was conducted in the absence of any commercial or financial relationships that could be construed as a potential conflict of interest.

## Publisher’s Note

All claims expressed in this article are solely those of the authors and do not necessarily represent those of their affiliated organizations, or those of the publisher, the editors and the reviewers. Any product that may be evaluated in this article, or claim that may be made by its manufacturer, is not guaranteed or endorsed by the publisher.

## Footnotes

1
http://www.geodata.cn/

2
https://search.asf.alaska.edu/
